# Gut-bone axis: mechanisms and intervention effects of Chinese botanical drugs in osteoporosis management

**DOI:** 10.3389/fphar.2026.1842067

**Published:** 2026-06-17

**Authors:** Shiyu Li, Hongyu Liu

**Affiliations:** 1 Rehabilitation Department, Central Hospital of Dalian University of Technology, Dalian Municipal Central Hospital, Dalian, Liaoning, China; 2 Department of Pharmacy, Central Hospital of Guangdong Prison, Guangzhou, Guangdong, China

**Keywords:** bone homeostasis, Chinese botanical drug, gut microbiota, gut-bone axis, osteoporosis

## Abstract

Osteoporosis (OP) is a systemic skeletal disorder characterized by decreased bone mass, impaired bone microarchitecture, and elevated fracture risk. With global population aging, OP has become a major public health burden worldwide. The gut-bone axis, a critical regulatory network connecting gut microbiota (GM) and bone metabolism, has emerged as a frontier in OP pathogenesis and intervention. GM dysbiosis disrupts bone homeostasis through metabolic, endocrine, and immune pathways, including short-chain fatty acids, estrogen/parathyroid hormone signaling, and Th17/Treg balance. Chinese botanical drugs (CBDs) exert unique advantages in OP management via holistic and multi-target effects, particularly by regulating GM to restore gut-bone axis balance. This review systematically elaborates the mechanisms by which GM contributes to OP, and summarizes advances in CBDs that regulate bone metabolism by remodeling GM composition, improving intestinal barrier function, and modulating gut-bone axis signaling. This work provides theoretical support for the clinical application and innovative research of CBDs, and lays a foundation for developing novel GM-targeted anti-OP therapeutic strategies.

## 1 Introduction

Osteoporosis (OP) is a systemic skeletal disorder characterized by reduced bone mass and impaired bone microarchitecture, leading to increased bone fragility and fracture susceptibility (Ye et al., 2025). Currently, the incidence of OP is increasing year by year, posing a significant threat to global health. According to statistics (Wu et al., 2021), an estimated 178 million new fragility fractures occurred worldwide in 2019, and this number is rising proportionally with population aging. Menopause and aging are the most common causes of OP. In addition, genetic susceptibility, lifestyle, and nutrition are also associated factors in its pathogenesis. Estrogen, parathyroid hormone (PTH), inflammatory cytokines, and vitamin D are key regulators in the bone remodeling process. Although clinical treatments (Sølling et al., 2020) targeting these regulators have demonstrated some efficacy, no current therapeutic approaches can fundamentally restore bone homeostasis. Therefore, exploring novel and effective therapeutic strategies for OP is of urgent clinical importance and social significance.

A variety of natural molecules derived from food and herbs have shown great potential in preventing and alleviating OP. Notable among these are phytoestrogens such as isoflavones (e.g., genistein and daidzein), which can mimic the bone-sparing effects of endogenous estrogen by preferentially binding to estrogen receptor-β, thereby inhibiting osteoclastogenesis while sparing reproductive tissues from potent estrogenic stimulation (Bitto et al., 2009). Prebiotic inulin-type fructans enhance calcium absorption and bone mineral density by stimulating short-chain fatty acid (SCFA)-producing gut commensals (Vandeputte et al., 2017). Other compounds like resveratrol and curcumin exert anti-osteoporotic effects primarily through their anti-inflammatory and antioxidant properties, suppressing the activation of NF-κB and MAPK signaling pathways that drive osteoclast differentiation, while the catalpol exhibits a dual action by both stimulating osteoblastogenesis via Wnt/β-catenin activation and suppressing osteoclast activity (Li, S. et al., 2025; Meyer et al., 2024; Zhu et al., 2019). A shared feature among many of these natural molecules is their poor oral bioavailability, which necessitates metabolic activation or modulation by the gut microbiota (GM) to exert their full biological effects. This pharmacokinetic bottleneck has increasingly focused attention on the GM as both a mediator and a direct therapeutic target in bone health.

Th GM, a complex and vast microbial community colonizing the human gastrointestinal tract, is composed of trillions of microorganisms, including bacteria, fungi, and archaea. The dominant bacterial phyla in the healthy human gut include *Bacteroidetes, Firmicutes, Actinobacteria, Proteobacteria,* and *Verrucomicrobia*, which exert profound influences on host physiology, ranging from the release of inflammatory cytokines and immune system homeostasis to intestinal nutrient absorption. In recent years, accumulating studies (Waldbaum et al., 2023) has shown that the abundance and composition of the GM in OP patients and animal models differ significantly from those of healthy controls. Moreover, the severity of bone loss correlates closely with the degree of GM dysbiosis, indicating that intestinal microbial imbalance may play a causal role in the pathogenesis of OP. Subsequent in-depth studies (Cao et al., 2024; Xiao et al., 2024) have further elucidated that the GM may regulate the relative activity and balance of osteoclasts and osteoblasts through its metabolic products, modulation of host endocrine function, regulation of intestinal barrier integrity and immune system homeostasis, as well as mediation of drug metabolism, thereby affecting host bone metabolism and contributing to the development and progression of OP. The discovery of the gut-bone axis and its multi-dimensional regulatory mechanisms has opened up a new research field and provided a novel theoretical basis for the prevention and treatment of OP.

Existing research on GM-targeted OP therapies has primarily focused on probiotics and prebiotics, while the potential of Chinese botanical drugs (CBDs)—with their long clinical application history, holistic regulation and multi-target characteristics—as effective GM modulators for OP intervention has long been overlooked. Modern pharmacological research (Fan et al., 2025; Li, J. et al., 2025) has confirmed that CBD formulations and their active metabolites can regulate bone metabolism by modulating the GM through specific molecular pathways, thereby exerting significant anti-OP therapeutic effects. This review discusses the mechanisms by which the GM mediates the onset and progression of OP (including regulation via GM metabolites, endocrine regulation, and immune regulation) and summarizes the CBD therapies currently used to prevent and treat OP by improving bone metabolism through these pathways. The objective is to promote the clinical application of CBD and provide a theoretical basis for its use in OP intervention.

## 2 Methods

This review was conducted based on a pre-established protocol, with a literature search performed on 5 January 2026, across four electronic databases (PubMed, Web of Science, Embase, and China National Knowledge Infrastructure (CNKI)) using the search terms “Gut microbiota” OR “Gut microflora” AND “Osteoporosis” OR “Bone loss” AND “Chinese botanical drug” OR “Chinese herbal medicine”; original studies investigating the regulatory role of GM in bone metabolism and the anti-osteoporotic effects of CBDs targeting GM were included, while irrelevant studies, reviews, conference abstracts, and articles with incomplete data were excluded. Two independent reviewers completed literature screening and data extraction, with disagreements resolved via consensus; a total of 262 records were initially identified, and 57 studies were finally included after deduplication and eligibility assessment, with detailed methodological procedures provided in Supplementary Material 1.

## 3 The gut-bone axis in the pathogenesis of OP

### 3.1 Alterations in GM profile in OP

The GM constitutes a complex microbial ecosystem and is widely recognized as the “second genome” of the human body (Sasso et al., 2023), exerting indispensable roles in host physiological homeostasis. Cohort studies (Campbell et al., 2023; Wang et al., 2023) verified that the core bacterial phyla of human GM include *Bacteroidetes, Firmicutes, Proteobacteria,* and *Actinobacteria,* with *Bacteroidetes* and *Firmicutes* accounting for over 90% of total microbial abundance. Under physiological conditions, GM maintains a dynamic ecological equilibrium, supporting intestinal barrier function, nutrient supply, and immune modulation—all essential for host homeostasis.

With advancing understanding of GM-mediated regulation of bone metabolism via microbial metabolites and immune signaling, the concept of the “gut-bone axis” was proposed (Zhang et al., 2024) and has become a central research focus in bone metabolism. Numerous studies have demonstrated that changes in GM abundance and composition directly participate in bone metabolism by regulating bone formation and resorption, with GM dysbiosis closely associated with OP development. Das et al. (Das et al., 2019) analyzed fecal microbial profiles in 181 elderly individuals with OP, osteopenia, or normal bone mass. Compared with healthy controls, OP and osteopenia patients showed significant GM compositional alterations, including markedly increased abundance of *Actinobacteria, Eggermannia, Clostridium*, and *Lactobacillus*; moreover, the decline in bone mineral density (BMD) was positively correlated with these microbial changes. Li C et al. (2019) performed high-throughput sequencing on fecal samples from 102 subjects with osteopenia or normal bone mass. The *Bacteroidetes phylum* was more abundant in the osteopenia group, while *Firmicutes* was more abundant in the normal group. Further correlation analysis showed that *Bacteroidetes* abundance was negatively correlated with BMD, whereas *Firmicutes* was positively correlated, suggesting that increased *Bacteroidetes* and decreased *Firmicutes* may be key features of GM dysbiosis leading to OP.

**FIGURE 1:**
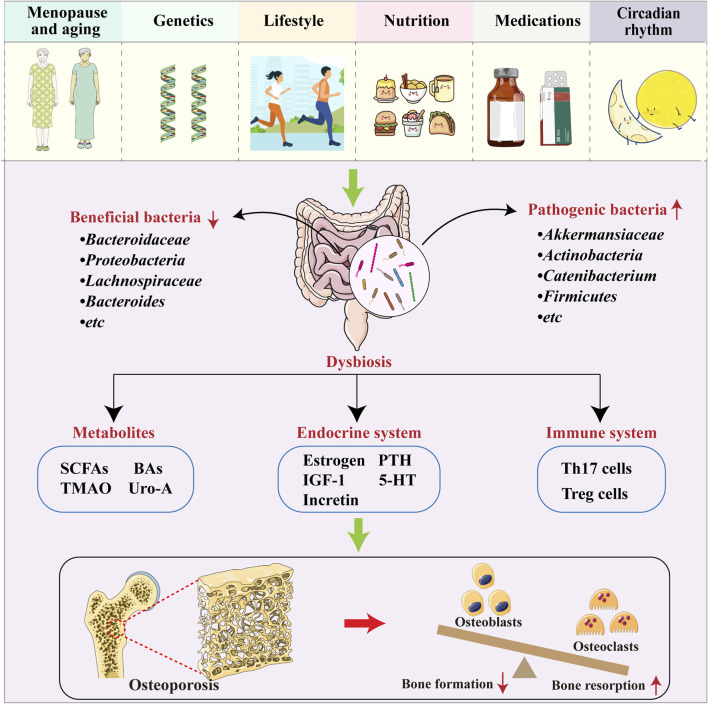
Schematic diagram illustrating the mechanistic link between GM dysbiosis and the pathogenesis of OP. A variety of factors can lead to GM dysbiosis (such as aging and menopause, genetics, lifestyle and medications), and further lead to the occurrence of OP. GM dysbiosis disrupts bone homeostasis through three interconnected regulatory axes: (1) Metabolite regulation: altered production of SCFAs, BAs, TMAO, and Uro-A directly or indirectly affects osteoclast and osteoblast activity; (2) Endocrine regulation: GM modulates PTH, estrogen, 5-HT, IGF-1, and incretins (GIP, GLP-1, GLP-2), thereby influencing bone remodeling; (3) Immune regulation: GM regulates the Th17/Treg cell balance, with Th17 cells promoting osteoclastogenesis via IL-17 and TNF-α, while Treg cells inhibit bone resorption via IL-4, IL-10, and TGF-β1. These pathways collectively contribute to bone loss and OP progression.

Animal studies also confirm a causal relationship between GM dysbiosis and OP. Germ-free (GF) mice exhibit fewer osteoclasts per bone surface area, along with higher volumetric BMD, bone mass fraction, and trabecular number (Sjögren et al., 2012). However, fecal microbiota transplantation from normal mice into GF mice for 4 weeks restored bone mass to normal levels, indicating that GM is an essential regulator of bone mass homeostasis. Additionally, Li et al. (2016) reported that estrogen-deficient GF mice did not exhibit significant bone loss compared with conventional mice, and administration of the probiotic *Lactobacillus rhamnosus* effectively prevented estrogen deficiency-induced bone loss in conventional mice. These findings further validate the close association between GM alterations and OP development, identifying GM as a key mediator of estrogen deficiency-induced bone loss. Figure 1 illustrates the mechanistic link between GM dysbiosis and OP pathogenesis.

### 3.2 Mechanisms underlying GM-mediated regulation of bone homeostasis

Based on these compositional changes, we further elaborate the mechanisms by which GM dysbiosis disrupts bone homeostasis. GM dysbiosis disrupts bone homeostasis and induces OP through three interconnected pathways: GM metabolite secretion, host endocrine modulation, and immune cell balance. These pathways form a complex regulatory network governing bone metabolism.

#### 3.2.1 GM metabolites involved in bone metabolism

**FIGURE 2:**
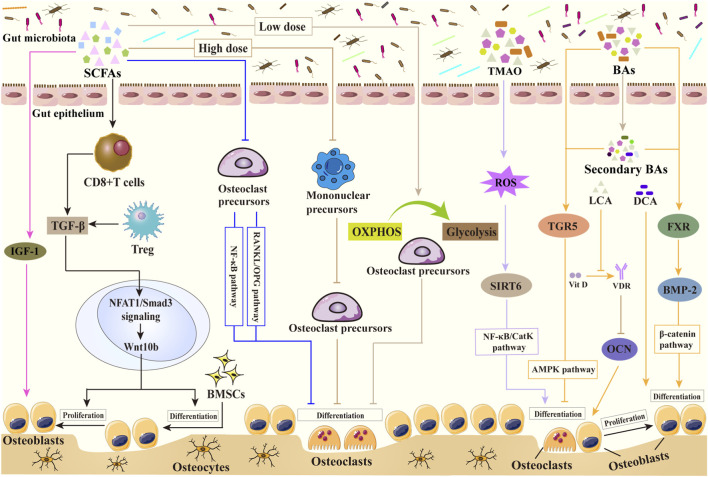
Overview of GM-derived metabolites and their effects on bone metabolism. GM interact with bone cells through multiple signaling pathways to modulate bone homeostasis: (I) SCFA-mediated regulation: SCFAs (produced by probiotics such as *Lactobacillus* and *Bifidobacterium*) bind to G protein-coupled receptors (GPCRs) on intestinal epithelial cells, promoting Treg cell proliferation and Wnt10b secretion by CD8^+^ T cells, which activates the Wnt/β-catenin pathway in BMSCs to enhance osteoblast differentiation; (II) BA signaling: Primary BAs are metabolized to secondary BAs (e.g., lithocholic acid, LCA; deoxycholic acid, DCA) by GM, which bind to FXR and TGR5 to regulate osteoclast/osteoblast activity via AMPK and NF-κB pathways; (III) VDR activation: Microbial metabolites modulate VDR signaling in osteoblasts, regulating the expression of OCN and RANKL/OPG to balance bone turnover; (IV) IGF-1 pathway: GM promotes hepatic IGF-1 secretion, which activates the PI3K/AKT pathway in BMSCs to enhance osteoblast proliferation and mineralization. Additionally, harmful metabolites (e.g., TMAO) induce ROS production, exacerbating osteoclast activation through the NFATc1/Smad3 pathway.

GM metabolites are secondary metabolites produced by GM in the intestine using dietary components and intestinal epithelial secretions as substrates. A healthy GM provides nutrients that the host cannot synthesize; conversely, GM dysbiosis increases harmful metabolites and exacerbates bone metabolic imbalance. Currently, GM metabolites associated with bone metabolism include short-chain fatty acids (SCFAs), tryptophan-related metabolites, bile acids (BAs), trimethylamine-N-oxide (TMAO), and Urolithin A (Uro-A). These metabolites regulate bone physiology by influencing cells involved in bone metabolism (Figure 2).

##### 3.2.1.1 SCFAs

SCFAs, the primary metabolites generated by GM fermentation of dietary fiber, mainly comprise acetic acid, propionic acid, and butyric acid, representing the most extensively studied GM metabolites in bone metabolism (Topping and Clifton, 2001). SCFAs directly act on bone cells and indirectly regulate bone metabolism through multiple pathways, playing a crucial role in maintaining bone mass homeostasis. Lucas et al. (Lucas et al., 2018) found that exogenous propionate and butyrate reduced expression of osteoclast-related genes TRAF6 and NFATc1 in mice, modulated osteoclast differentiation, and inhibited bone resorption *in vitro* and *in vivo*, thereby significantly increasing bone mass. Further studies revealed that SCFAs inhibit osteoclast differentiation by inducing metabolic reprogramming in osteoclast precursor cells, enhancing glycolysis and oxidative phosphorylation, leading to downregulation of key osteoclastogenic transcription factors. De Martinis et al. (2020) reported that butyrate may regulate bone formation and resorption balance by inducing histone acetylation and modulating miRNA expression in bone cells. In addition, Tyagi et al. (2018) demonstrated that butyrate stimulates Treg cell production in the intestine and bone marrow of mice, thereby promoting expression of the anabolic bone factor Wnt10b in bone marrow CD8^+^ T cells, which further activates the Wnt pathway to promote bone formation (Kobayashi et al., 2016). This provides a direct immunologically-mediated pharmacological pathway: the bacterial metabolite butyrate acts as a histone deacetylase (HDAC) inhibitor, promoting Foxp3+ Treg cell differentiation, which in turn secretes Wnt10b to directly stimulate osteoblastogenesis on bone surfaces.

SCFAs also regulate bone metabolism by stimulating the synthesis of insulin-like growth factor-1 (IGF-1) in the liver and adipose tissue (Chen et al., 2022). IGF-1 is a key growth factor that promotes osteoblast proliferation and differentiation. Chen et al. (2007) reported that sodium butyrate stimulated osteogenic differentiation of mesenchymal stem cells (MSCs) and upregulated the expression of the osteogenic transcription factor Runx2 by activating the extracellular signal-regulated kinase (ERK) signaling pathway. Furthermore, SCFAs lower intestinal pH, prevent calcium-phosphate complexation, enhance intestinal calcium absorption and utilization (Chen et al., 2019), and inhibit receptor activator of nuclear factor κB ligand (RANKL)-induced osteoclast formation by suppressing osteoclast-related gene expression. A clinical study (Whisner et al., 2014) in adolescents f demonstrated that *Bacteroides*, *Bifidobacterium*, and *Fusobacterium* in GM degrade dietary fiber into SCFAs, reduce local intestinal pH, and significantly increase calcium absorption and BMD. Additionally, butyrate repairs intestinal mucosal and villus structure, increases intestinal absorptive surface area, and further enhances calcium absorption and utilization. Collectively, SCFAs effectively alleviate OP by regulating osteoclast and osteoblast formation and activity through multiple pathways, serving as key beneficial metabolites of the gut-bone axis.

##### 3.2.1.2 Tryptophan-related metabolites

Tryptophan is an essential aromatic amino acid and a biosynthetic precursor for various microbial and host metabolites that regulate multiple physiological functions including bone metabolism. L-kynurenine and indole-3-propionic acid (IPA) are two major GM-derived tryptophan metabolites that exert opposite effects on bone metabolism.

The divergent effects of these metabolites are mediated through distinct pharmacological mechanisms on bone cells. L-kynurenine is a major tryptophan metabolite whose concentration in the body is closely regulated by the GM. Animal studies (Refaey et al., 2017) have shown that oral gavage or intraperitoneal injection of L-kynurenine in mice leads to significant bone loss, characterized by a marked decrease in bone volume fraction, trabecular bone thickness, and other bone microstructural parameters, as well as a significant increase in serum levels of the osteoclastic marker RANKL. In addition, L-kynurenine can induce age-related genetic changes in bone marrow stem cells, such as reduced expression of HDAC3 and NcOR1 genes and increased expression of lipid storage-related genes CideC and Plin1. Furthermore, Pierce et al. (2020) found that L-kynurenine inhibits mitochondrial respiration in mouse osteoblasts *in vitro*, disrupting cellular energy metabolism and suppressing bone formation. Specifically, L-kynurenine acts as a direct inhibitor of cytochrome c oxidase, impairing the electron transport chain and reducing ATP production required for osteoblast matrix synthesis. Clinical studies (Kim et al., 2019; Zhang et al., 2009) have further confirmed a close association between L-kynurenine and bone metabolism: bone marrow L-kynurenine levels correlate positively with age and negatively with total femoral bone mass, and patients with higher bone marrow L-kynurenine concentrations exhibit elevated serum levels of osteoclastic markers such as TRAP and RANKL. These findings indicate that the age-related increase in L-kynurenine in the body may be an important factor contributing to increased bone fragility in the elderly.

IPA, a tryptophan metabolite exclusively produced by the GM, exerts beneficial effects on bone metabolism. Studies (Lee et al., 2025) have shown that probiotic supplementation can increase serum IPA levels, prevent intestinal inflammation in obese mice, and improve intestinal barrier function by upregulating the expression of tight junction proteins (ZO-1, Occludin, Claudin-5). Behera et al. (2021) found that IPA can enhance the expression of the mitochondrial transcription activator TFAM by increasing the binding of the histone demethylase Kdm6b and reducing the binding of H3K27me3 to the TFAM promoter, thereby promoting osteoblast differentiation. In addition, IPA can suppress the expression of the Toll-like receptor 4 receptor (TLR4) and prevent endotoxin-induced osteoblast dysfunction, further protecting bone formation. This established a causal pathway from a specific GM metabolite (IPA) to a defined nuclear target and a functional bone cell outcome.

##### 3.2.1.3 BAs

BAs are amphipathic molecules primarily produced by hepatocytes (primary BAs) and further metabolized into secondary BAs by *Firmicutes* in the intestine. BAs and their receptors (Farnesoid X receptor (FXR), G protein-coupled bile acid receptor 1 (TGR5)) play an important role in the regulation of bone metabolism by influencing the differentiation and activity of osteoblasts and osteoclasts through various mechanisms.

FXR and TGR5 are the two main BA receptors, which are widely expressed in bone cells and play a key role in regulating carbohydrate, lipid, and energy metabolism (Ocaña-Wilhelmi et al., 2021). Study using metabolomic and gut microbial profiling in postmenopausal osteoporosis (PMO) models have shown altered BA profiles and elevated BA levels in OP conditions, accompanied by significant shifts in GM composition (Wen et al., 2020). Excessive BAs may exert antibacterial effects on intestinal bacteria, leading to changes in GM richness and composition, which in turn disturb intestinal BA metabolism and serum BA concentrations, ultimately affecting osteoclast activity and contributing to bone loss. Li Z et al. (2019) demonstrated that TGR5 inhibits osteoclast differentiation via the AMP-activated protein kinase (AMPK) signaling pathway, and the dual activation of TGR5 and FXR effectively improves estrogen deficiency-induced bone loss in mice, suggesting that BA receptors are potential therapeutic targets for OP.

In addition, secondary BAs can act as ligands for the vitamin D receptor (VDR) and modulate vitamin D-mediated bone metabolism (Ruiz-Gaspà et al., 2010). Vitamin D binds to VDR in osteoblasts and significantly increases the expression of CYP24A, osteocalcin, and RANKL mRNA, exerting a direct regulatory effect on bone metabolism. However, treatment of osteoblasts with secondary BAs significantly reduces the expression of these genes, indicating that secondary BAs exert an inhibitory effect on osteoblasts and bone formation.

##### 3.2.1.4 TMAO

TMAO is a key GM metabolite produced by the microbial metabolism of dietary choline and L-carnitine. Elevated plasma TMAO levels are associated with increased production of pro-inflammatory cytokines, which in turn promote osteoclast formation and exacerbate bone loss. However, the role of TMAO in bone metabolism may be context-dependent. Zhou et al. (2019) found that after 6 months of a weight-loss diet, type 2 diabetes patients showed significantly reduced plasma TMAO and its precursor L-carnitine, accompanied by decreased spinal and hip BMD, suggesting that TMAO may protect against bone density loss during weight loss in diabetic patients. The dual mechanism of TMAO in bone metabolism requires further clarification.

##### 3.2.1.5 Uro-A

Uro-A is a natural GM metabolite from ellagitannins and ellagic acid, with confirmed anti-inflammatory and antioxidant properties (Abdelazeem et al., 2021). Recent studies show that Uro-A also exerts significant anti-OP effects by inhibiting osteoclast formation. Tao et al. (2021) found that Uro-A can attenuate osteoclast formation by downregulating the inflammatory cascade. Further mechanistic studies (Tao et al., 2022) have shown that in RANKL-induced osteoclastogenesis *in vitro*, Uro-A inhibits bone resorption by enhancing the autophagy of bone marrow macrophages and suppresses osteoclast formation by inhibiting the mitogen-activated protein kinase (MAPK) signaling pathway, thereby exerting a protective effect on bone mass.

#### 3.2.2 Endocrine regulation via the gut-bone axis

**FIGURE 3:**
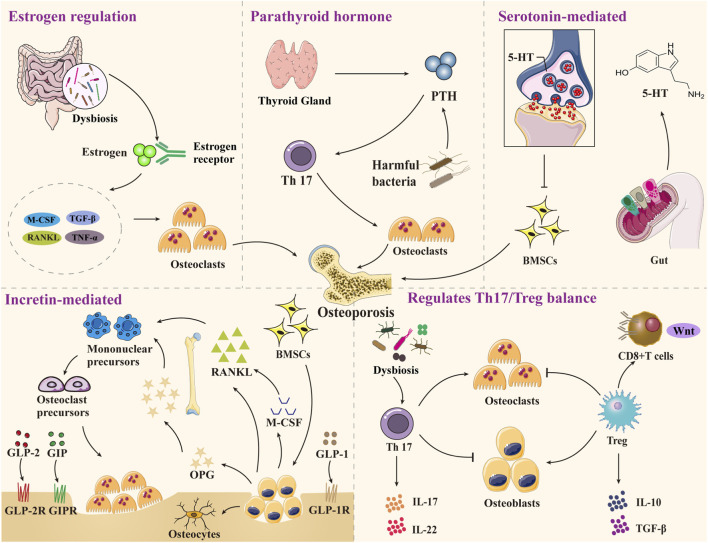
Endocrine and immune mechanisms of the gut-bone axis in OP. GM dysbiosis disrupts bone homeostasis by modulating endocrine factors and immune cell balance. Endocrine regulation: GM and its metabolites (e.g., SCFAs) influence the secretion and activity of key hormones: parathyroid hormone (PTH) – PTH-dependent bone formation requires butyrate to expand Treg cells and activate Wnt10b; estrogen–GM regulates estrogen levels and the RANKL/RANK/OPG pathway; serotonin (5-HT) – GM controls enteric 5-HT synthesis via TPH-1, and enteric 5-HT inhibits bone formation while brain-derived 5-HT promotes osteogenesis; insulin-like growth factor-1 (IGF-1) – SCFAs stimulate hepatic IGF-1 production, which activates Wnt/β-catenin signaling in osteoblasts; incretins (GIP, GLP-1, GLP-2) – GM modulates incretin secretion, affecting bone formation and resorption. Immune regulation: SCFAs promote the differentiation of anti-inflammatory Treg cells, which secrete IL-4, IL-10, and TGF-β1 to inhibit osteoclast formation. Conversely, GM dysbiosis favors pro-inflammatory Th17 cells, which produce IL-17 and TNF-α, promoting osteoclastogenesis and bone resorption via NF-κB and MAPK pathways.

Skeletal growth and bone metabolism are highly dependent on the host endocrine system and its regulatory factors. IGF-1, estrogen, PTH, and 5-hydroxytryptamine (5-HT) are well-characterized key endocrine factors regulating bone homeostasis (Kverka and Stepan, 2025). Accumulating evidence has demonstrated that the GM can modulate the secretion and activity of these endocrine factors, thereby indirectly regulating bone metabolism (Figure 3). Therefore, investigating the crosstalk between the GM and the endocrine system in OP has become a current research focus, and identifying corresponding preventive and therapeutic strategies is of great significance for OP management.

##### 3.2.2.1 PTH

PTH is a key bone metabolism regulator with dual effects: low-dose intermittent PTH promotes bone formation, while high-dose continuous PTH induces bone resorption. Notably, both effects require GM and its metabolite mediation. Yu et al. (2020) found that in mouse models of primary and secondary hyperparathyroidism, intestinal overgrowth of segmented filamentous bacteria increases intestinal TNF + T cells and Th17 cells, which migrate to the bone marrow and induce bone loss. Further studies (Li J et al., 2020) revealed that the bone-forming effect of PTH depends on butyrate, a key SCFA produced by GM. Butyrate promotes PTH-induced increases in bone marrow Treg cells, which upregulate the osteogenic Wnt ligand Wnt10b via bone marrow CD8^+^ T cells, thereby activating the Wnt-dependent bone formation pathway. Microbial butyrate production is thus a necessary cofactor for PTH-mediated bone anabolism.

##### 3.2.2.2 Estrogen

Estrogen is the primary hormone regulating bone metabolism in females. It improves bone metabolism mainly by inhibiting osteoclast formation and activation while stimulating osteoblast activity. Estrogen deficiency, a major cause of PMO, enhances osteoclast activity by activating the RANKL/RANK/OPG pathway, leading to excessive bone resorption and loss. Concurrently, estrogen deficiency also causes damage to the intestinal mucosal barrier, increases intestinal permeability, and promotes the release of pro-inflammatory cytokines (TNF-α, IL-1β), which further upregulate the number and activity of osteoclasts and exacerbate OP progression.

Basic and clinical studies demonstrate that estrogen-deficient OP patients exhibit significant GM compositional changes and reduced microbial diversity (Chen L et al., 2021). Ma et al. (2020) observed severe GM dysbiosis in ovariectomized (OVX) rats with OP, with a significantly increased *Firmicutes/Bacteroidetes* ratio. *Ruminococcus*, *Clostridium*, and *Coprobacter* were positively correlated with bone loss, while *Bacteroidetes* were negatively correlated. Restoring GM exerts beneficial effects on PMO: supplementation with *Lactobacillus brevis* in OVX mice exerts anti-osteoporotic effects by reducing bone resorption (Yu et al., 2022). Mechanistically, *L. brevis* regulates the RANKL/OPG ratio by inhibiting pro-inflammatory osteoclastogenic cytokines and suppresses osteoclast precursor differentiation via the TRAF6/NF-κB/NFATc-1 pathway after RANKL binding to RANK. These findings indicate that GM regulates estrogen deficiency–induced bone loss by modulating intestinal inflammatory responses and bone metabolism–related signaling pathways, and GM modulation represents a potential therapeutic strategy for PMO.

##### 3.2.2.3 5-HT

5-HT is a biogenic amine that plays a dual role in bone metabolism, with enteric 5-HT (produced by intestinal enterochromaffin cells) inhibiting bone formation and brain-derived 5-HT promoting osteogenesis and inhibiting bone resorption. The GM is a key regulator of enteric 5-HT secretion: *Streptococcus* and *Escherichia coli* in the intestines of healthy mice can upregulate serum levels of enteric 5-HT (Sjögren et al., 2012), and spore-forming anaerobic bacteria can also modulate 5-HT levels in the serum and colonic feces of mice (Yano et al., 2015). After transplanting spore-forming anaerobes from healthy human intestines into GF mice, the 5-HT levels in the serum and colonic feces of GF mice increased significantly compared with the control group, confirming the regulatory effect of the GM on enteric 5-HT secretion.

Chronic alcohol consumption can alter the GM composition, leading to increased secretion of enteric 5-HT, which in turn significantly inhibits the osteogenic activity and bone mineralization of bone marrow-derived stem cells (BMSCs) and induces OP (Liu et al., 2022). Park et al. (2018) demonstrated that intracellular signaling via the 5-HT receptor 6 (5-HT6R) is associated with RhoA GTPase activation and contributes to osteoclast maturation; conversely, inhibition of 5-HT6R-mediated RhoA GTPase signaling protects against bone loss in OVX mice. In addition, the catalytic activity of tryptophan hydroxylase (Tph), the rate-limiting enzyme in 5-HT synthesis, is a key marker for 5-HT production. Yadav et al. (2010) found that oral administration of Tph-1 inhibitor (the initial enzyme in 5-HT synthesis) for 6 weeks can increase the number of osteoblasts, bone formation rate, and serum osteocalcin levels in OVX mice, significantly improving OP symptoms. Kode et al. (2012) further demonstrated that enteric 5-HT inhibits osteoblast proliferation by acting on the skeletal transcription factor FOXO1 and transcription activators, thereby reducing bone formation. Collectively, enteric 5-HT is an important mediator of microbial regulation of bone homeostasis, and targeting the GM-5-HT axis is a novel potential strategy for OP treatment.

##### 3.2.2.4 IGF-1

IGF-1 is a multifunctional growth factor that exerts endocrine, paracrine, and autocrine effects on bone tissue and plays a crucial role in bone formation and maturation by promoting the proliferation and osteogenic differentiation of BMSCs via the Wnt/β-catenin signaling pathway (Feng and Meng, 2021). Both the GM and its key metabolite SCFAs can influence bone metabolism by modulating host IGF-1 levels (Yan and Charles, 2018). Schwarzer et al. (2016) found that GF mice have significantly lower IGF-1 levels compared with conventional mice. Yan et al. (2016) confirmed that antibiotic-induced GM depletion reduces serum IGF-1 levels in mice and inhibits bone formation, while supplementation with SCFAs to antibiotic-treated mice restores both IGF-1 levels and bone mass to pre-treatment levels. These findings indicate that the GM regulates bone metabolism by promoting IGF-1 synthesis, and the GM-IGF-1 axis is an important regulatory pathway of the gut-bone axis.

##### 3.2.2.5 Incretin

Incretins are a class of gut-derived hormones that stimulate insulin secretion in a glucose-dependent manner, including glucose-dependent insulinotropic peptide (GIP) and glucagon-like peptides (GLP-1, GLP-2). Accumulating evidence has shown that incretins also play an important role in bone metabolism regulation. GIP binds to receptors on osteoblasts, increases the expression of type I collagen-related genes, stimulates collagen synthesis and maturation, enhances alkaline phosphatase activity, and promotes bone formation (Liu H et al., 2024). In addition, GIP binds to receptors on pre-osteoclasts, reduces osteoclast maturation and activity, thereby decreasing bone resorption rates. GLP-1 enhances insulin secretion by pancreatic β-cells, and insulin exerts a positive effect on bone formation; simultaneously, GLP-1 enhances calcitonin secretion by thyroid C cells, thereby reducing bone resorption. Previous studies (Yadav et al., 2010) have shown that the GM can stimulate enterochromaffin cells to secrete 5-HT, which then crosses the intestinal barrier into the bloodstream and reduces GLP-1 secretion, thereby decreasing osteoblast formation and inhibiting bone formation. GLP-2, another member of the incretin family, improves intestinal blood flow, promotes the proliferation of intestinal epithelial cells, and reduces intestinal permeability. In aged rats, aging leads to a decrease in the expression of intestinal tight junction proteins, increased intestinal permeability and enhanced chronic inflammatory responses, which in turn activate the RANK/RANKL/OPG signaling pathway to promote osteoclast proliferation and bone resorption (Ren et al., 2014). Notably, Wu et al. (2018) found that GLP-2 can modulate the GM in aged rats and reduce the abundance of the *Spirochaetales phylum*, which is associated with intestinal inflammation. However, further research is needed to determine whether GLP-2 can improve OP by modulating the GM and intestinal barrier function.

In summary, GM and its metabolites regulate bone metabolism by modulating the secretion and activity of multiple endocrine factors (PTH, estrogen, 5-HT, IGF-1, incretins), thereby maintaining bone homeostasis. GM dysbiosis disrupts endocrine regulation of the gut-bone axis, leading to bone metabolic disorders and OP.

#### 3.2.3 Immune modulation as a core link in the gut-bone axis

The immune system is a key bridge connecting the GM and bone metabolism, and immune cell imbalance is an important mechanism by which GM dysbiosis induces OP. Th17 and Treg cells, two subsets of CD4^+^ T cells with opposing functions, play a crucial role in the immune regulation of bone metabolism: Th17 cells promote osteoclast formation and bone resorption, while Treg cells inhibit osteoclast activity and protect bone mass. The GM regulates bone metabolism by modulating the Th17/Treg balance in the intestine and bone marrow, representing a key immune regulatory mechanism of the gut-bone axis.

##### 3.2.3.1 Th17 cells

Th17 cells are a pro-inflammatory T cell subset that plays a critical role in the pathogenesis of OP. Ciucci et al. (2015) demonstrated by flow cytometry and gene expression analysis that bone marrow Th17 cells in osteoporotic mice can produce large amounts of IL-17 and TNF-α, which are key pro-inflammatory cytokines promoting osteoclast formation. Liu et al. (2019) found that IL-17 can increase NF-κB expression via the PKC-ERK/MAPK pathway, and as an upstream signal of the NLRP3 inflammasome, NF-κB activation increases the expression of the NLRP3 inflammasome, adhesion molecules, and E-selectin, elevating levels of various pro-inflammatory factors and thereby inducing metabolic bone disease. TNF-α not only directly induces osteoclastogenesis but also reduces bone formation by increasing RANKL expression in the body through the regulation of MAPK phosphorylation. Postler and Ghosh (2017) found that TNF-α expression increases significantly in OVX mice, while supplementation with *Lactobacillus reuteri* significantly reduces TNF-α and RANKL expression and decreases the number of osteoclasts, suggesting that the GM can regulate Th17 cell-mediated bone resorption. Furthermore, GM dysbiosis may lead to the massive migration of intestinal Th17 cells into the bone marrow, where they recruit osteoclast precursors and induce excessive osteoclast formation and bone loss (Ibeagha-Awemu et al., 2021). Studies have shown that Th17 cells are most abundant in the lamina propria of the small intestine, and bacteria such as *Bifidobacterium* and *Enterobacteriaceae* in the GM are sufficient to induce Th17 cell differentiation in animal models. Although there is no definitive evidence linking Th17 cell production directly to the GM, the close correlation between Th17 cells and OP progression and the GM-dependent differentiation of Th17 cells suggest that the GM is an important regulator of Th17 cell-mediated bone metabolism.

##### 3.2.3.2 Treg cells

Treg cells are an anti-inflammatory T cell subset that plays a critical role in suppressing autoimmunity and maintaining immune tolerance in the body. Treg cells also accumulate in large numbers in the lamina propria of the small intestine, a phenomenon that is closely associated with the enrichment of *Clostridia* and *Bacteroides* in the GM and their production of SCFAs (Sefik et al., 2015). SCFAs promote the proliferation and differentiation of intestinal Treg cells through two main mechanisms: ① binding to the G protein-coupled receptor (GPR43) on colonic epithelial cells to induce Treg cell proliferation; ② inhibiting the activity of histone deacetylases (HDACs) to promote Treg cell differentiation. Sefik et al. (Sefik et al., 2015) found that the abundance of Treg cells in the mesentery of GF mice is significantly reduced, while transplantation of GM (including *Clostridium*, *Bacteroides*, *Bifidobacterium,* and *Lactobacillus*) promotes the generation of Treg cells in the mesentery of GF mice, confirming the regulatory effect of the GM on Treg cell development.

Treg cells play a key role in bone metabolism regulation by secreting anti-inflammatory cytokines that inhibit osteoclast formation (Lam et al., 2021). Treg cells can secrete TGF-β1, IL-4, and IL-10, among which TGF-β1 promotes osteogenesis by inhibiting osteoclast expression, increasing Wnt1 protein production, and activating the Smad2/3 signaling pathway via the TGF-α receptor complex, thereby promoting the migration and differentiation of osteoblast precursors and downregulating osteoclast formation. IL-4 and IL-10 can inhibit RANKL-induced osteoclast differentiation and promote the methylation of the lncRNA MEG3 to suppress its expression. Huang et al. (2022) found that Treg cells isolated from FoxP3 transgenic mice suppress osteoclast formation by producing IL-4 and IL-10 when reinfused into the peripheral blood of mice, confirming the anti-osteoporotic effect of Treg cells.

In summary, Th17 and Treg cells exert opposing effects on bone mass: Th17 cells promote osteoclast formation and bone resorption by secreting pro-inflammatory cytokines (IL-17, TNF-α), while Treg cells inhibit osteoclast activity by secreting anti-inflammatory cytokines (TGF-β1, IL-4, IL-10). The GM can regulate the dynamic balance between Th17 and Treg cells in the intestine and bone marrow through its metabolites (e.g., SCFAs), thereby increasing the secretion of anti-inflammatory cytokines, inhibiting osteoclast proliferation and differentiation, reducing bone resorption, and maintaining bone mass homeostasis. GM dysbiosis disrupts the Th17/Treg balance, leading to excessive bone resorption and the development of OP.

## 4 Anti-osteoporotic mechanisms of CBDs via targeting the gut-bone axis

### 4.1 Natural chemical metabolites

Natural chemical metabolites derived from CBDs constitute the primary material basis for their anti-OP effects, including alkaloids, flavonoids, lignans, saponins, and polysaccharides. These metabolites exert significant GM-modulating effects and anti-OP activity by regulating the gut-bone axis through multiple pathways. The anti-OP effects and mechanisms of typical CBD metabolites are summarized in Table 1.

**TABLE 1:** Natural chemical metabolites for the gut-bone axis to relieve OP.

Natural chemical metabolites	Resource	Experiment model	Dose	Duration time	Minimal effective dose	Negative/Positive control (NC/PC)	Effects on GM	Potential anti-osteoporotic activity	Potential limitations/Controversies	References
Berberine	*Coptis chinensis Franch*	OVX-Periodontitis Rat	120 mg/kg/d (administered by gavage)	7 weeks	120 mg/kg	NC: ‐; PC: ‐	Enriches *Blautia, norank_f_Bacteroidales_S24-7_*group, *Roseburia*; elevates fecal butyrate; restores *Allobaculum, Turicibacter*	Inhibits mesial/distal bone resorption; increases BV/TV, decreases Tb.Sp; reduces osteoclasts, increases osteoblasts	Only validated in periodontitis models; no evidence in primary OP; no causality confirmation; no human trials	Jia et al. (2019), Yue et al. (2019)
Puerarin	*Pueraria montana (Lour.) Merr*	OVX rats	Low dose (50 mg/kg/day) or high dose (100 mg/kg/day) (administered by gavage)	14 weeks	50 mg/kg	NC: ‐; PC: ‐	Increases Shannon index; normalizes F/B ratio; upregulates *Lactobacillus*, *Bifidobacterium*; downregulates *Desulfovibrio*; enriches SCFA synthesis	Raises femoral BMD; increases Tb.N, Tb.Th, BV/TV, decreases Tb.Sp; reduces CTX-1, TRAcP-5b; preserves ZO-1, Occludin	Only tested in OVX rats; cannot fully mimic human postmenopausal OP; no long-term safety or clinical data	Li B et al. (2020)
Lignans	*Sambucus williamsii Hance*	OVX rats	Low dose (140 mg/kg/d), high dose (280 mg/kg/d) (administered by gavage)	10 weeks	140 mg/kg	NC: ‐; PC: Teriparatide (1.8 μg/kg) group (intramuscular injection)	Elevates *Actinobacteria;* upregulates *Adlercreutzia, Collinsella; decreases [Eubacterium]_coprostanoligenes, Ruminococcaceae_UGC-014*	Increases femur/tibia BMD; elevates Tb.N, Conn.D, reduces Tb.Sp, SMI; increases OCN, decreases CTX-1; downregulates colonic TPH1	Mechanisms based on correlation; no causal verification; only rat studies; no human dosage data	Xiao et al. (2018), Xiao et al. (2022)
Loganin	*Cornus officinalis Siebold & Zucc*	1. *In vitro*: MC3T3-E1 cells 2. *In vivo*: OVX mice	1. *In vitro*: 0.01, 0.05, 0.1 μM 2. *In vivo*: 5 mg/kg/d, 10 mg/kg/d (gastric administration)	1. *In vitro*: 7 d 2. *In vivo*: 12 weeks	1. *In vitro*: 0.05 μM 2. *In vivo*: 5 mg/kg	NC: ‐; PC: ‐	Increases *Bacteroidetes, Firmicutes*; reduces *Proteobacteria pathogens*; enriches *Lactobacillus, Bifidobacterium, Blautia; lowers Desulfovibrio, Bacteroides*	Promotes MC3T3-E1 osteogenesis; increases BMD, BV/TV, Tb.N, Tb.Th, decreases Tb.Sp; reduces CTX-1, TRAcP-5b; inhibits NF-κB, preserves ZO-1, Occludin	Causal link between GM shift and anti-OP effect unproven; GM biotransformation unclear; no human trials	XIE et al. (2024)
Oleanolic Acid	*Ligustrum lucidum Ait. and Eclipta prostrata (L.) L.*	OVX mice	10 mg/kg/d (intraperitoneal injection)	12 weeks (once every other day)	10 mg/kg	NC: ‐; PC: ‐	Elevates *Actinobacteriota*; upregulates *Staphylococcus, Coriobacteriaceae_UCG-002*; decreases *Odoribacter*	Increases femoral BMD, BV/TV, reduces Tb.Sp; lowers β-CTX, TRACP5b, P1NP; decreases TNF-α, IL-6, LPS, increases IL-10	Only correlative evidence; no GM depletion validation; i.p. Injection inconsistent with clinical use; no long-term safety	MA et al. (2023)
Total flavonoids *Eucommia ulmoides Oliv. leaves*	*Eucommia ulmoides Oliv*	OVX rats	200 mg/kg/d (gastric administration)	13 weeks	200 mg/kg	NC: ‐; PC: Estradiol group (0.208 mg/kg/d) (gastric administration)	Balances *Bacteroidetes/Firmicutes*; increases *Campylobacterota*; reduces *Pr*evotella, elevates *Muribaculaceae*	Raises femoral BMD, BV/TV, Tb.N, Tb.Th, lowers Tb.Sp, SMI; attenuates OVX-induced weight gain; regulates bone metabolism	Only in OVX model; core active components unclear; no human PK or clinical data	Zhang, Y. et al. (2022)
Astragalus polysaccharides (APS)	*Astragalus membranaceus (Fisch.) Bunge*	SD rat (Dexamethasone-induced)	50, 150, and 250 mg/kg/d (administered by gavage)	8 weeks	50 mg/kg	NC: ‐; PC: ‐	Upregulates *Ruminococcaceae*, *Alloprevotella;* downregulates *Blautia*, *Lactobacillus, Akkermansia*; restores amino acid/carbohydrate metabolism	Restores femoral BMD; increases Tb.Ar, BV/TV, Tb.N, Tb.Th, decreases Tb.Sp; reduces ACP5, TNF-α, IL-2	Only in GC-induced OP; not suitable for primary OP; indirect mechanism; no human safety data	Liu et al. (2020)
Water extract of *Epimedium brevicornu Maxim*	*Epimedium brevicornu Maxim*	OVX rats	0.81 g/kg/d (administered by gavage)	12 weeks	0.81 g/kg	NC: ‐; PC: ‐	Increases *Muribaculaceae, Lactobacillus*; decreases *Firmicutes_unclassified*; enriches *Ruminococcaceae_UGC-014, Candidatus_Saccharimonas*	Increases trabecular number; reduces fractures and fat vacuoles; upregulates Runx2; reverses femur/lumbar BMD loss	Complex mixture; active ingredients unclear; no FMT for axis causality; no human bone trials	LIN et al. (2023)
Water extract of *Ligustrum lucidum* *Ait*	*Ligustrum lucidum Ait*	OVX rats	3.5 g/kg/d (administered by gavage)	14 weeks	3.5 g/kg	NC: ‐; PC: Estradiol valerate group (0.1 mg/kg/day) (administered by gavage)	Elevates cecal SCFAs; increases *Bifidobacterium*, normalizes F/B ratio	Raises BMD, BV/TV, Tb.N, Tb.Th, reduces Tb.Sp, SMI; enhances maximum load and flexural strength; increases PINP, decreases CTX-1; promotes calcium absorption	Only in OVX rats; cannot represent human OP; key GM-regulating components unknown; no long-term observation	Chen, B. et al. (2021)
Morinda officinalis How polysaccharides	*Morinda officinalis How*	1. *In vitro*: rat BMSCs 2. *In vivo*: OVX mice	1. *In vitro*: 0, 10, 20, 40 μg/mL 2. *In vivo*: 100 mg/kg/day (gastric administration)	1. *In vitro*: 7 days 2. *In vivo*: 12 weeks	1. *In vitro*: 20 μg/mL 2. *In vivo*: 100 mg/kg/d	NC: ‐; PC: ‐	Balances *Bacteroidetes/Firmicutes*; enriches *Lactobacillus, Bifidobacterium*, *Akkermansia*; lowers *Desulfovibrio, Bacteroides*	Promotes BMSCs osteogenic differentiation; increases BMD, BV/TV, Tb.N, decreases Tb.Sp; reduces CTX-1, TRAcP-5b; inhibits NF-κB, preserves ZO-1, Occludin	Only animal data; no human validation; intestinal metabolism unclear; no long-term toxicity data	Zhang, C. et al. (2022)

Berberine, the main component of *Coptis chinensis Franch.*, is an isoquinoline alkaloid with broad-spectrum antibacterial activity. Recent studies have shown that berberine also exerts a significant anti-OP effect by modulating the GM. Yue et al. (2019) found that berberine alleviated collagen-induced arthritis in rats by regulating butyrate metabolism, increasing the number of butyrate-producing bacteria, reducing nitrate synthesis, and stabilizing physiological colonic hypoxia, thereby mitigating periodontal bone loss. Jia et al. (2019) demonstrated that berberine enhanced intestinal barrier function and modulated host immunity by regulating GM, and significantly ameliorated periodontal bone loss in mice. Given that SCFAs, intestinal mucosal barrier integrity, and host immunity are all key regulatory factors of the gut-bone axis, berberine may positively regulate bone metabolism by altering GM composition, increasing beneficial metabolite (butyrate) production, and modulating host immune responses. However, direct evidence for berberine’s anti‐OP effect via the gut-bone axis remains indirect and largely speculative, as these findings were obtained in periodontal bone loss and arthritis models rather than primary or PMO models. Extrapolation of these mechanisms to systemic OP requires validation in dedicated OP models.

Puerarin, an isoflavone compound extracted from *Pueraria montana (Lour.) Merr.*, exhibits estrogen-like effects and is a classic CBD for the treatment of PMO. Li B et al. (2020) found that puerarin reversed GM dysbiosis induced by estrogen deficiency in OVX rats, increased intestinal SCFA content, repaired damaged intestinal mucosa, reduced colonic epithelial permeability, and decreased pro-inflammatory factor (TNF-α, IL-6, IL-1β) release, thereby improving the bone microenvironment and exerting anti-OP effects. Notably, puerarin exerts bone-protective effects primarily by modulating the gut-bone axis rather than through direct estrogen-like effects, as it reverses estrogen deficiency-induced bone loss without binding to estrogen receptors. Fecal microbiota transplantation from puerarin-treated to untreated OVX rats partially recapitulated the bone-protective phenotype, providing functional evidence for a gut-bone axis-mediated mechanism. This finding provides a novel theoretical basis for the clinical application of puerarin in PMO treatment.

Lignans are the main active components of *Sambucus williamsii Hance*, a plant used in folk medicine to treat bone disorders. Xiao et al. (2018) found that the lignan-rich fraction from *S. williamsii Hance* can downregulate 5-HT levels in RBL-2H3 cells with high TPH-1 expression. Further studies (Xiao et al., 2022) demonstrated that lignans inhibited colonic TPH-1 protein expression, increased the relative abundance of antibacterial bacteria, and reduced serum 5-HT levels in OVX rats, thereby exerting anti-OP effects. As GM is a key regulator of intestinal TPH-1 expression and 5-HT secretion, these results suggest that the anti-OP effect of lignans on enteric 5-HT is GM-mediated, and the GM-5-HT axis is a key pathway for lignans to exert bone-protective effects.

Loganin, the primary active iridoid glycoside isolated from *Cornus officinalis Siebold & Zucc.*, possesses multiple pharmacological effects, including anti-inflammatory, immunomodulatory, and bone metabolism-regulating properties (DAI et al., 2022). Xie et al. (2024) found that loganin can increase femoral BMD, bone volume/total volume (BV/TV), trabecular bone number, and serum procollagen type I N-terminal propeptide (P1NP) levels in OVX mice, while reducing serum C-terminal cross-linked peptide (CTX) and tartrate-resistant acid phosphatase levels, exerting a significant anti-OP effect. Mechanistically, loganin can increase the abundance of unclassified bacteria in the *Muribaculaceae* family and decrease the abundance of *Lactobacillus*, thereby improving GM dysbiosis in OVX mice, inhibiting osteoclast formation, and reducing bone resorption. However, the current evidence for loganin’s anti-OP mechanism via the gut-bone axis is primarily associative. Although GM modulation and improved bone parameters were concurrently observed, the direct causal link between specific GM changes and the anti-OP effects—as well as the precise molecular mediators involved—remains to be elucidated through functional studies, such as fecal microbiota transplantation or targeted metabolite intervention.

Oleanolic acid, a triterpenoid compound widely present in kidney-tonifying CBDs such as *Ligustrum lucidum Ait.* and *Eclipta prostrata (L.) L.*, possesses potential anti-inflammatory and antioxidant effects and significant natural advantages in OP prevention and treatment (Wang et al., 2025). Ma et al. (2023) found that oleanolic acid can increase BMD, BV/TV, and serum IL-10 levels in OVX mice, while decreasing serum P1NP, β-collagen degradation products, TRACP5b, TNF-α, and IL-6 levels. In terms of GM modulation, oleanolic acid increases the abundance of Actinobacteria, *Staphylococci*, *Bacteroides,* and *Enterobacteriaceae (UCG-002)*, while reducing the abundance of pro-inflammatory *Bacteroides*. These findings indicate that oleanolic acid can slow bone loss and increase bone mass by reducing intestinal inflammatory responses, improving intestinal permeability, and regulating GM structure, thereby exerting a therapeutic effect on OP. Nevertheless, the evidence linking oleanolic acid’s anti-OP effects to the gut-bone axis remains largely correlative. Although GM structural modulation and improved bone parameters were observed concurrently in OVX mice, definitive functional evidence—such as demonstrating that the anti-OP effect is abolished upon GM depletion or that GM transfer from oleanolic acid-treated mice confers bone protection—is currently lacking.

Astragalus polysaccharides are polysaccharides extracted from *Astragalus membranaceus (Fisch.) Bunge*, which possesses various biological activities. Studies (Hu et al., 2023) have shown that they are effective against OP. Liu et al. (2020) found that Astragalus polysaccharides can significantly improve the intestinal microbiota composition in rats with a dexamethasone-induced model by upregulating probiotic communities such as *Lactobacillus* and *Bacteroides*, downregulating pathogenic bacteria such as *Clostridium* and *Prevotella*, significantly reducing the production of acid phosphatase 5 and pro-inflammatory cytokines IL-2 and TNF-α, inhibiting osteoclast formation, and improving OP. However, it should be acknowledged that this study employed a dexamethasone-induced rat model rather than a primary OP model, and the gut-bone axis mechanistic evidence—while suggestive—remains indirect. The observed GM changes and anti-OP effects are correlative, and whether the GM modulation is a primary mediator or merely a secondary concomitant phenomenon requires further investigation using approaches such as GM depletion or fecal microbiota transplantation. Other studies (Van Wijngaarden et al., 2013; Villa et al., 2017) have shown that the GM exerts anti-OP effects by promoting the differentiation of osteoblasts into bone cells through the synthesis and metabolism of vitamins B and K, inhibiting osteoclast formation, and mediating the calcification process of osteocalcin. Therefore, while studying the effects of CBDs on GM composition, the impact of the microbiota on drug absorption and metabolism in the host—which may influence the anti-OP efficacy of these medications—also warrants attention.

*Epimedium brevicornu Maxim.*, a classic CBD for tonifying the liver and kidneys and strengthening the tendons and bones, is widely used in OP treatment. Icariin is the main active flavonoid component of *E. brevicornu Maxim.*, and the water extract of *E. brevicornu Maxim.* also exhibits significant anti-OP effects. Liu et al. (2018) found that the water extract of *E. brevicornu Maxim.* can promote bone formation through mechanisms involving neuropeptide Y and vasoactive intestinal peptide, thereby exerting an anti-osteoporotic effect. Lin et al. (2023) demonstrated that the water extract of *E. brevicornu Maxim.* can improve the femoral microstructure and collagen fiber number in OVX rats, increase Runx2 levels, reduce the abundance of the *Firmicutes phylum,* and increase the abundance of the *Bacteroidetes* and *Proteobacteria phyla* in the GM, suggesting that its anti-OP mechanism is closely related to GM modulation. Furthermore, Wang et al. (2022) found that icariin can significantly upregulate the metabolism of BAs, amino acids, and fatty acids in OVX rats, thereby elevating estrogen levels and producing an anti-osteoporotic effect. Nevertheless, the mechanistic evidence linking Epimedium water extract and icariin specifically to the gut-bone axis in OP remains largely correlative. While GM alterations and metabolite changes have been observed alongside improvements in bone parameters in OVX rats, the causal involvement of the gut-bone axis—as opposed to direct pharmacological actions on bone cells or other systemic pathways—has not been definitively established and warrants further targeted investigation. Since the GM is a key regulator of BA and estrogen metabolism, these findings indicate that icariin may influence estrogen metabolism by regulating the GM, which is a potential mechanism for its anti-OP effect.

*Eucommia ulmoides Oliv.* has effects such as tonifying the kidneys and invigorating qi, as well as strengthening tendons and bones; it can significantly increase femoral bone mass and bone density in normal rats (XIE et al., 2022). The lignans and total flavonoids from *E. ulmoides Oliv.* are the main active components exerting bone-protective effects. Zhao et al. (2020) found that the lignans from *E. ulmoides Oliv.* can improve the relative abundance of *Bacteroidetes* and *Bifidobacteria* in rapidly aging mice, increase the concentrations of SCFAs in feces and serum, thereby increasing BMD and reducing bone loss. Zhao et al. (2023) suggested that *E. ulmoides Oliv.* extract can modulate GM composition to promote SCFA production, inhibit osteoclast formation and improve bone metabolism. These studies indicate that *E. ulmoides Oliv.* maintains skeletal health by regulating the GM and promoting the production of microbial metabolites (SCFAs), which is an important mechanism for its anti-OP effect.

### 4.2 CBD formulations

Classic CBD formulations are developed based on traditional Chinese medicine (TCM) theory and exhibit multi-component, multi-target, and holistic regulatory characteristics. In recent years, numerous studies have demonstrated that classic CBD formulations exert significant anti-OP effects by modulating GM and the gut-bone axis, with more comprehensive and stable therapeutic effects than single active metabolites. The anti-OP effects and GM-modulating mechanisms of typical classic CBD formulations are summarized as follows (Table 2).

**TABLE 2:** Anti-osteoporotic effects of CBD formulations via regulating the gut-bone axis.

Formulations	Formulation composition	Experiment model	Dose	Duration time	Negative/Positive control (NC/PC)	Effects on GM	Potential anti-osteoporotic activity	Potential limitations/Controversies	References
Gegen Qinlian Decoction (GGQLD)	*Pueraria montana (Lour.) Merr., Scutellaria baicalensis Georgi, Coptis chinensis Franch.,* and *Glycyrrhiza uralensis Fisch*	HFD/STZ rats	25 g/kg/day (administered by gavage)	12 weeks	NC: ‐; PC: Metformin group (250 mg/kg/d) (gastric administration)	Elevates Shannon index; enriches SCFA-producing bacteria; reduces opportunistic pathogens; reverses GM dysbiosis	Upregulates Occludin/claudin-1; lowers inflammatory cytokines; improves glucose metabolism; protects pancreatic function	Only in diabetic OP; not for primary/postmenopausal OP; no causality or human GM–bone trials	Tian et al. (2021), WANG et al. (2021)
Tenghuang Jiangu pill (THJGP)	*Rehmannia glutinosa (Gaetn.) Libosch. ex Fisch. et Mey., Davallia trichomanoides Blume, Cistanche deserticola Ma, Epimedium brevicornu Maxim.,* and *Spatholobus suberectus Dunn, etc*	OVX rats	0.15, 0.3, 0.6 g/kg/day (administered by gavage)	8 weeks	NC: ‐; PC: Xianlinggubao capsule group (0.10 g/kg/d) (gastric administration)	Normalizes F/B ratio; enriches *Lactobacillus/Akkermansia/Bacter*oides; suppresses harmful bacteria; upregulates SCFA metabolism	Increases BMD/BV/TV/Tb.Th; regulates bone markers; inhibits IL-6; activates Wnt/β-catenin pathway	Only in OVX rats; cannot simulate complex human OP; core components unclear; no human long-term data	ZHANG (2024)
Xianling Gubao capsule (XLGBC)	*Epimedium brevicornu Maxim., Dipsacus asperoides C. Y. Cheng et T .M. Ai., Psoralea corylifolia Linn., Salvia miltiorrhiza Bunge,* and *Rehmannia glutinosa (Gaetn.) Libosch. ex Fisch. et Mey., etc*	OVX rats	1 g/kg/d (administered by gavage)	3 months	NC: ‐; PC: ‐	Reduces *Firmicutes, elevates Bacteroidetes*; lowers F/B ratio; enriches beneficial bacteria; enhances drug metabolism	Improves lipid metabolism; increases bioavailability of active ingredients; regulates microbiota-metabolite-bone axis	Animal results not directly translatable; no large RCT with GM endpoints; long-term GM impact unknown	Tang et al. (2021)
Yishen Zhuanggu decoction (YSZGD)	*Dipsacus asperoides C. Y. Cheng et T .M. Ai., Psoralea corylifolia, Achyranthes bidentata Blume, Eucommia ulmoides Oliv.,* and *Panax notoginseng (Burkill) F. H. Chen ex C. H. Chow (Ratio: 1:1:1:1:2)*	Glucocorticoid-induced OP (GIOP) mice (dexamethasone phosphate-indued)	1, 4 g/kg/d (by gavage)	6 weeks	NC: ‐; PC: ‐	Restores GM diversity; eliminates pathogenic bacteria; enriches beneficial *Enterococcus*	Downregulates Osterix mRNA; alleviates glucocorticoid-induced bone loss; protects gut-bone axis	Only for GC-induced OP; hypothetical mechanism; no causal proof; no human efficacy/safety data	CAO et al. (2021)
Jiangu granule (JGG)	*Davallia trichomanoides Blume, Epimedium brevicornu Maxim.,* and *Cornus officinalis Siebold & Zucc., etc*	OVX rats	Equivalent dose of crude drug: 2 g/kg/d (administered by gavage)	6 weeks, 12 weeks (in two phases)	NC: ‐; PC: ‐	Lowers F/B ratio; enriches SCFA-producing bacteria; elevates fecal SCFAs; upregulates ZO-1/Occludin	Increases BMD/TMD/BV/TV; balances bone turnover; regulates Treg/Th17; modulates OPG/RANKL pathway	Only in OVX rats; no humanized GM model; long-term effects unconfirmed in humans; optimal clinical cycle unclear	Sun et al. (2022)
Zhuanggu Zhitong capsule (ZGZTC)	*Epimedium brevicornu Maxim., Ligustrum lucidum Ait., Psoralea corylifolia Linn., Davallia trichomanoides Blume,* and *Achyranthes bidentata Blume, etc*	OVX rats	1.944 g/kg/d (administered by gavage)	12 weeks	NC: ‐; PC: Estradiol valerate (EV) group (0.09 mg/kg/d) (administered by gavage)	Optimizes *Firmicutes/Bacteroidetes* ratio; enriches *Romboutsia*; reduces pro-inflammatory taxa	Improves bone microstructure; upregulates Occludin/ZO-1; regulates IL-17/TGF-β; exerts estrogen-like anti-OP effect	Limited translational value; drug–GM interaction unclear; no combined GM–bone clinical trials	ZHANG et al. (2023)
Shengu granule (SGG)	*Astragalus membranaceus (Fisch.) Bunge, Salvia miltiorrhiza Bunge, Angelica sinensis, Eucommia ulmoides Oliv.,* and *Cuscutachinensis Lam., etc*	OVX rats	12 g/kg/d (administered by gavage)	12 weeks	NC: ‐; PC: ‐	Enriches SCFA-producing bacteria; decreases pro-inflammatory microbiota; elevates fecal SCFAs; repairs intestinal barrier	Increases bone density and trabecular quality; regulates bone markers; activates Wnt10b/β-catenin pathway	Mechanisms speculative; only in OVX model; no human data; potential GM disturbance risk unknown	LI et al. (2024)
Bushen Jianpi formula (BSJPF)	*Codonopsis pilosula (Franch.) Nannf., Atractylodes macrocephala Koidz.,Dioscorea polystachya Turcz., Rehmannia glutinosa (Gaetn.) Libosch. ex Fisch. et Mey.,* and *Cornus officinalis Siebold & Zucc., etc*	OVX rats	2.98 g/kg/d (gastric administration)	12 weeks	NC: ‐; PC: ‐	Repairs intestinal villi; upregulates Cldn3; inhibits intestinal inflammatory factors	Increases BMD/Ct.Th; reduces osteoclasts; downregulates C-FOS/CTSK; alleviates bone marrow inflammation	No FMT causality confirmation; multi-component synergy unclear; no large-scale human trials	TAN et al. (2024)
Bushen Huoxue decoction (BSHXD)	*Rehmannia glutinosa (Gaetn.) Libosch. ex Fisch. et Mey., Eucommia ulmoides Oliv., Cuscutachinensis Lam.,* and *Cistanche deserticola Ma, etc*	OVX rats	0.94 g/kg/d (gastric administration)	8 weeks	NC: ‐; PC: estradiol valerate (0.0184 mg/kg) (gastric administration), probiotics (30.85 mg/kg) (gastric administration)	Regulates *Firmicutes/Proteobacteria* ratio; repairs intestinal mucosa; inhibits TLR4/MyD88/NF-κB pathway	Increases ALP/OCN; decreases TRACP-5b; repairs bone trabeculae; suppresses bone tissue inflammatory signaling	Causal direction of GM–bone regulation unproven; only OVX model; no human evidence; long-term GM effects unknown	Han et al. (2024)

Gegen Qinlian Decoction (GGQLD), composed of *P. montana (Lour.) Merr.*, *Scutellaria baicalensis Georgi*, *C. chinensis Franch.* and *Glycyrrhiza uralensis Fisch.*, is a classic CBD formulation with the effects of relieving exterior symptoms and clearing internal heat. It has demonstrated good clinical efficacy in the treatment of diabetes and lipid metabolism disorders, and long-term glucose and lipid metabolism abnormalities are often accompanied by bone metabolic disorders. Wang and Lan (2020) found that GGQLD not only treats diabetes but also increases BMD, bone calcium content, and maximum load-bearing capacity in diabetic OP rats. Mechanistically, GGQLD can improve the structure and diversity of the GM, inhibit pathogenic bacteria, increase intestinal SCFA concentration, and modulate host glucose and lipid metabolism through the GM, thereby exerting a positive therapeutic effect on diabetes-induced OP (Tian et al., 2021; WANG et al., 2021). However, it must be emphasized that the evidence for GGQLD’s anti-OP effect via the gut-bone axis is indirect, as the primary study model was diabetic rats rather than a primary or PMO model. The extrapolation from diabetes-associated bone loss to systemic OP should be made with caution. The observed benefits on bone parameters in the context of diabetes may be secondary to improved glucose metabolism and reduced systemic inflammation, and a specific gut-bone axis-mediated anti-OP mechanism in non-diabetic OP remains to be demonstrated.

Tenghuang Jiangu pill (THJGP), a classic CBD formulation with the effects of tonifying the kidneys, promoting blood circulation, and relieving pain, is widely used in the treatment of various orthopedic conditions. Zhang et al. (2024) found that THJGP can enhance the richness and diversity of the GM in OVX rats, increase the levels of *Bacteroides*, *Lactobacillus*, *Akkermansia,* and reduce the levels of *Ruminococcus*, *Firmicutes,* and the *Curcumae* genus, thereby improving intestinal homeostasis after ovariectomy. THJGP exerts its anti-OP effects through lipid metabolism regulation: it promotes the differentiation of BMSCs into osteoblasts, inhibits their differentiation into adipocytes, suppresses the production of fatty acid-like compounds, and modulates the intestinal microenvironment, thereby improving bone metabolism in OP rats.

Xianling Gubao Capsule (XLGBC), a clinical first-line CBD formulation for OP treatment, has the effects of nourishing the liver and kidneys, promoting blood circulation and unblocking meridians, and strengthening tendons and bones. It is commonly used to treat OP caused by liver and kidney deficiency and blood stasis obstructing the meridians. Tang et al. (2021) found that XLGBC can alter the GM structure in OVX rats, accelerate drug deglycosylation reactions, facilitate the absorption and metabolism of active ingredients such as icariin, psoralen, and isopsoralen, and promote the growth of *Bacteroides*, *Prevotella,* and the probiotic *Lactobacillus*. XLGBC can also regulate lipid and BA metabolism by modulating the GM, thereby improving bone metabolism and exerting an anti-OP effect. These findings confirm that the GM plays a significant role in the absorption and metabolism of XLGBC, and XLGBC exerts its anti-OP effect by modulating the GM and the gut-bone axis.

Yishen Zhuanggu decoction (YSZGD) is composed of *Dipsacus asperoides C. Y. Cheng et T .M. Ai.*, *Psoralea corylifolia*, *Achyranthes bidentata Blume*, *E. ulmoides Oliv.*, and *Panax notoginseng*. It has the effects of tonifying the kidneys and liver, and promoting blood circulation to remove blood stasis. Studies (Mo et al., 2025) have shown that the active components in *A. bidentata Blume, E. ulmoides Oliv.*, and *P. notoginseng* have a beneficial regulatory effect on the GM (CAO et al., 2021). Cao et al. (2021) found that YSZGD can alter the GM structure in glucocorticoid-induced OP mice, inhibit the growth of pathogens such as *Aeromonas*, downregulate the mRNA levels of the osteoblast-specific gene osterix in renal tissue, and improve bone metabolism. It is hypothesized that YSZGD treats osteopenia by downregulating osterix mRNA levels in renal tissue and inhibiting the proliferation of intestinal pathogenic bacteria, thereby restoring the balance of the gut-bone axis. Nonetheless, this hypothesized gut-bone axis mechanism is based on a glucocorticoid-induced OP model with observations of concurrent GM changes and bone improvement. The direct causal link between GM modulation (inhibition of *Aeromonas*), renal osterix expression, and bone protection remains speculative and requires experimental verification—for example, through selective elimination or reconstitution of specific bacterial taxa and assessment of the downstream effects on bone metabolism.

Jiangu granule (JGG), composed of 10 herbal ingredients including *Davallia trichomanoides Blume, E. brevicornu Maxim.*, and *C. officinalis Siebold & Zucc.,* etc., is a commonly used CBD formulation in clinical practice for the treatment of PMO. Sun et al. (2022) found that JGG ameliorated GM dysbiosis caused by estrogen deficiency, restored GM abundance, regulated bone-related immune cytokines through the GM-SCFAs-Treg/Th17 axis, effectively reduced bone loss, and improved bone metabolism in OVX rats. Long-term intervention (12 weeks) of JGG was more effective than short-term intervention (6 weeks) in improving bone quality, modulating GM, and promoting SCFA production, suggesting that JGG exerts favorable therapeutic effects on PMO through the gut-bone axis.

Zhuanggu Zhitong capsule (ZGZTC), with the effects of tonifying the liver and kidneys, strengthening the tendons and bones, and promoting blood circulation to relieve pain, can increase BMD and delay the onset and progression of OP. Zhang et al. (2023) found that ZGZTC can lower serum IL-17 levels, increase TGF-β levels, significantly improve intestinal inflammation, reduce the abundance of the *F. phylum,* and increase the abundance of the *B. phylum* in OVX rats. ZGZTC can also interact with estrogen to regulate bone metabolism, and its anti-OP mechanism is related to modulating GM structure, reducing the production of inflammatory factors, and improving intestinal inflammation, thereby restoring the bone microstructure in OP rats.

Shengu granule (SGG), a compound CBD formulation with the effects of dispelling dampness, strengthening the spleen, and fortifying the tendons and bones, demonstrates significant efficacy in patients with OP characterized by spleen deficiency and dampness retention. Li et al. (2024) found that SGG can significantly improve intestinal mucosal inflammatory infiltration and ulcers in OVX rats, increase intestinal SCFA secretion, elevate serum bone alkaline phosphatase (ALP) and osteocalcin (OCN) levels, and reduce serum P1NP levels. SGG can also increase BMD, improve bone microstructure, restore GM diversity, and repair intestinal mucosal damage. Its anti-OP mechanism may involve correcting GM imbalance, activating the Wnt/β-catenin signaling pathway via SCFAs, and thereby improving bone metabolism. However, this proposed mechanism—GM correction leading to SCFA-mediated Wnt/β-catenin activation—remains speculative and based on correlative evidence. Direct experimental demonstration that SGG-induced SCFA production activates the Wnt/β-catenin pathway in osteoblasts in a GM-dependent manner, and that this pathway is causally responsible for the observed bone protection, has not yet been provided.

Bushen Jianpi formula (BSJPF), derived from modifications to the Lu Jiao pills, has the effects of tonifying the kidneys and replenishing essence, as well as strengthening the spleen and benefiting qi. Its efficacy in preventing and treating OP has been clinically validated over many years, and it also offers unique advantages in maintaining GM balance (Liao et al., 2024). Wang, (2023) found that the BSJPF promotes the prevention and treatment of OP by increasing *Lactobacillus abundance*, inhibiting *Clostridium* growth, upregulating growth hormone and IGF-1 levels, activating the PI3K/AKT signaling pathway, and upregulating osterix protein expression, thereby promoting osteoblast proliferation and differentiation and increasing bone formation. Tan et al. (2024) also confirmed that BSJPF can repair the damaged intestinal barrier, reduce the activation of intestinal immune inflammatory cells and the production of chemokines, inhibit osteoclastic activity, and improve bone loss in OVX rats, thereby exerting a therapeutic effect on OP.

Bushen Huoxue Decoction (BSHXD), derived from *The Great Compendium of Traumatology*, is a classic CBD formulation with the effects of tonifying the kidneys, promoting blood circulation, and strengthening the bones. Modern pharmacological studies (LIU, S.-H. et al., 2024) have shown that BSHXD exerts anti-inflammatory and antioxidant effects and promotes cartilage repair through multiple mechanisms. A study by Han et al. (2024) demonstrated that BSHXD can restore GM diversity, reduce colonic inflammatory infiltration, and lower serum levels of IL-1β, IL-6, and TNF-α. Concurrently, the expression of TLR4, myeloid differentiation factor 88 (MyD88), and NF-κB p65 protein expression in both colonic and femoral tissues, indicating that (BSHXD can alleviate the body’s inflammatory response by improving the structure of the gut microbiota, inhibit the TLR4/MyD88/NF-κB signaling pathway, thereby modulating the level of inflammation in the bone microenvironment and influencing bone metabolism, thus achieving the effect of preventing and treating OP. However, while this study links GM modulation with suppression of the TLR4/MyD88/NF-κB pathway in both colon and bone, the directional causality—whether GM improvement drives the anti-inflammatory bone protection or vice versa—has not been established. The concurrent regulation of this pathway in both tissues could reflect parallel pharmacological effects of BSHXD components rather than a sequential gut-bone axis mechanism, and functional studies controlling for GM status are needed to resolve this question.

## 5 Discussion and future perspectives

OP is defined as a systemic bone disease characterized by a decrease in bone mass and deterioration of bone microstructure, leading to increased bone fragility and a heightened risk of fractures (Ye et al., 2025). OP-related fractures are a leading cause of disability and mortality in the elderly, and the rising incidence of osteoporotic fractures driven by global population aging has drastically increased healthcare expenditures, posing an acute challenge to worldwide healthcare systems. Current clinical anti-OP therapies are categorized into bone resorption inhibitors, bone formation promoters, and dual-action agents (Table 3), yet these drugs suffer from inherent limitations, including suboptimal efficacy, severe adverse reactions, high costs, and restricted clinical applicability (Golledge and Thanigaimani, 2022), leaving a critical gap for therapies that can fundamentally restore bone metabolic homeostasis.

**TABLE 3:** Current drugs for OP treatment and their limitations.

Drug category	Common drugs	Mechanism	Limitations	References
Anti-resorptive Drugs	Bisphosphonates (e.g., Alendronate: 70 mg/week or 10 mg/day orally; Zoledronic Acid: 5 mg/year intravenously)	Bind to bone hydroxyapatite, inhibit osteoclast activity and induce apoptosis, reducing bone resorption	Gastrointestinal adverse reactions; long-term use increases risks of jaw osteonecrosis and atypical femoral fracture; contraindicated in severe renal insufficiency, hypocalcemia and esophageal diseases	Ahdi et al. (2023)
	SERMs (e.g., Raloxifene: 60 mg/day orally)	Tissue-selective estrogen receptor binding, inhibits bone resorption and reduces fracture risk	Hot flashes, venous thrombosis risk; contraindicated in venous thrombosis history, severe liver dysfunction and pregnancy	Adomaityte et al. (2008)
	Calcitonin (e.g., Salmon Calcitonin: 200 IU/day s.c./i.m. or nasal spray)	Directly inhibits osteoclasts, relieves bone pain and slightly increases short-term bone density	Long-term drug resistance; limited long-term efficacy; not a first-line agent	Tang et al. (2014)
	Denosumab: 60 mg every 6 months s.c	Binds RANKL, inhibits osteoclast formation/activation, strongly reducing bone resorption	Infection and hypocalcemia risk; long-term use affects bone turnover; contraindicated in hypocalcemia and severe immune deficiency	Bird et al. (2024)
	Etidronate Disodium: 200 mg twice daily orally (2 weeks on, 10 weeks off)	Binds bone minerals, inhibits osteoclast-mediated resorption and reduces bone turnover	Requires cyclic administration; may affect bone mineralization; contraindicated in severe renal insufficiency and hypocalcemia	Heaney and Saville (1976)
	Teriparatide: 20 μg/day s.c	PTH receptor agonist, promotes osteoblast proliferation, stimulates bone formation and reduces fractures	Max 2-year course (osteosarcoma risk); hypercalcemia; high cost; contraindicated in osteosarcoma/bone metastases	Harper et al. (2007)
Bone-forming Drugs	Romosozumab: 210 mg/month s.c. for 12 months	Binds sclerostin, promotes bone formation and inhibits resorption, dual-regulating bone metabolism	Cardiovascular event risk; injection site reactions; long-term safety unconfirmed; contraindicated in cardiovascular disease	Asadipooya and Weinstock (2019)
	Strontium Ranelate: 2 g/day orally (before bedtime)	Dual effects: inhibits osteoclasts and promotes osteoblasts, increasing bone density	Gastrointestinal/skin reactions; venous thrombosis risk; contraindicated in severe renal insufficiency and thrombosis	Ali et al. (2020)
Dual-action Drugs	Bazedoxifene/Conjugated Estrogens: 1 tablet/day orally	Combination inhibits bone resorption; bazedoxifene counteracts estrogen-induced uterine/breast stimulation	Hot flashes, vaginal bleeding; contraindicated in hormone-dependent tumors and venous thrombosis	Duggan and McKeage (2011)

In recent years, mounting evidence (Feng et al., 2024; Li et al., 2021; Liu H et al., 2024; Zheng et al., 2025) has unraveled a tight association between gut microbiota (GM) and host bone metabolism, with GM compositional dysbiosis emerging as a pivotal inducer of bone metabolic abnormalities and subsequent OP development. The GM modulates host metabolic, immune, and inflammatory homeostasis and acts as a core regulator of bone metabolism by shaping immune responses and osteoclast formation, thereby governing bone quality. It exerts direct and indirect regulatory effects on bone mass via modulating host metabolism, endocrine hormone levels, and immune system function, while GM-derived metabolites serve as reliable readouts of microbial impacts on bone metabolism, offering novel potential targets for OP prevention and treatment. Specifically, a healthy GM and sufficient SCFAs are indispensable for intestinal barrier maintenance and repair, whereas GM dysbiosis impairs intestinal mucosal integrity and increases permeability. This barrier disruption allows microbe-associated molecular patterns to enter the systemic circulation, which either directly bind to osteoclasts or indirectly perturb immune homeostasis—particularly the Th17/Treg balance—ultimately triggering bone loss (Guo et al., 2023). Moreover, hormones (especially estrogen) act as key regulators linking GM and bone metabolism, and the GM can exert detrimental effects on bone health by upregulating enteric 5-HT production and TPH-1 transcription. Collectively, targeting the GM has become a novel and promising therapeutic strategy for OP management.

**FIGURE 4:**
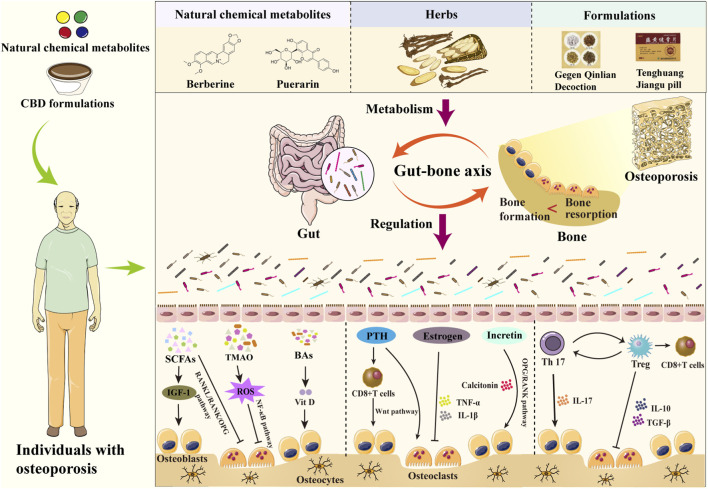
Anti-osteoporotic mechanisms of CBDs targeting the gut-bone axis. CBDs (natural chemical metabolites and classic formulations) exert anti-OP effects by restoring GM homeostasis. Natural metabolites such as berberine, puerarin, lignans, loganin, oleanolic acid, icariin, and polysaccharides from *Astragalus membranaceus (Fisch.) Bunge, Epimedium brevicornu Maxim., Ligustrum lucidum Ait.*, and *Morinda officinalis How* modulate GM composition-increasing SCFA-producing bacteria (e.g., *Lactobacillus, Bifidobacterium, Blautia, Roseburia*), reducing pathogenic taxa (e.g., *Desulfovibrio, Clostridium*), repairing intestinal barrier (upregulating ZO-1, occludin), and suppressing systemic inflammation (↓TNF-α, IL-6, LPS). Lower panel: Classic CBD formulations (Gegen Qinlian Decoction, Tenghuang Jiangu Pill, Xianling Gubao Capsule, Yishen Zhuanggu Decoction, Jiangu Granule, Zhuanggu Zhitong Capsule, Shengu Granule, Bushen Jianpi Formula, Bushen Huoxue Decoction) similarly reshape GM, promote SCFA production, regulate Th17/Treg balance, and activate bone-forming pathways (e.g., Wnt/β-catenin, OPG/RANKL) while inhibiting osteoclastogenesis. These actions collectively restore bone metabolic homeostasis and alleviate OP.

Against this backdrop, CBDs have emerged as a promising new avenue for OP prevention and treatment via targeted GM modulation, and this review systematically clarifies the core regulatory mechanisms of the gut-bone axis in OP pathogenesis while comprehensively summarizing the therapeutic potential of CBDs as GM modulators, yielding several novel findings that integrate CBD with modern osteology, microbiology, and immunology. A key novel discovery is that GM dysbiosis functions as a causal driver rather than a secondary byproduct of bone metabolic imbalance, with microbial metabolites (SCFAs, tryptophan metabolites, BAs), endocrine crosstalk (the estrogen-PTH-5-HT axis), and immune cell polarization (the Th17/Treg balance) forming an interconnected regulatory network that modulates osteoclast-osteoblast coupling. Notably, CBDs exert anti-OP effects predominantly through targeted remodeling of the GM ecosystem—a distinct mechanism that differentiates them from conventional anti-OP drugs and embodies the holistic regulatory advantage of CBD for chronic metabolic bone diseases. Another critical novel finding is that both single CBD bioactive metabolites (berberine, puerarin, icariin, loganin) and classic CBD formulations (Xianling Gubao Capsule, Bushen Huoxue Decoction, Jiangu granule) share a conserved gut-bone axis-mediated anti-OP pathway: they correct OP-associated GM dysbiosis (e.g., reducing the *Firmicutes/Bacteroidetes* ratio and enriching SCFA-producing probiotics), repair intestinal barrier integrity, and inhibit systemic low-grade inflammation, thereby restoring bone metabolic homeostasis (Figure 4). Additionally, this review identifies that GM-mediated biotransformation of CBDs (e.g., intestinal bacterial deglycosylation of icariin and psoralen) is a key step for their bioavailability and therapeutic efficacy, establishing a bidirectional “CBD-GM” interaction loop that provides a modern scientific explanation for the clinical efficacy of TCM formulations. Specific GM biomarkers (e.g., *Lactobacillus, Bifidobacterium*, and SCFA-producing *Blautia* and *Roseburia*) associated with bone mass maintenance are also identified, offering new targets for the predictive assessment and precision intervention of OP.

Despite these advances, current research on CBD-mediated gut-bone axis regulation for OP has notable limitations that impede basic research translation and novel therapy development. ① most studies focus on single CBD metabolites or formulations in rodent OP models (OVX, glucocorticoid-induced), lacking exploration of multi-component CBD synergism—the core of TCM holistic therapy—and the molecular mechanisms of inter-component interactions in gut-bone axis regulation. Critically, the vast majority of CBD evidence cited in this review derives from OVX or glucocorticoid-induced rodent models. While these models recapitulate certain aspects of postmenopausal or glucocorticoid-induced bone loss, they inherently fail to capture the multifactorial, chronic, and heterogeneous nature of human OP, which arises from cumulative interactions among aging, genetic predisposition, dietary patterns, lifestyle factors, polypharmacy, and comorbidities. This over-reliance on reductionist animal models constitutes a fundamental translational barrier; ② animal models fail to replicate the complexity of human OP (influenced by aging, genetics, lifestyle and microbial diversity), with a scarcity of large-scale human cohort studies and clinical trials validating GM-OP correlations and CBD efficacy/safety in diverse populations. The robust anti-OP effects observed with CBDs in these simplified rodent systems should not be directly extrapolated to the heterogeneous elderly patient population without rigorous human validation. specifically, to date, no published human trials have simultaneously assessed both gut microbiota endpoints (e.g., metagenomic sequencing, SCFA profiling) and bone outcomes (e.g., BMD, bone turnover markers) for the CBD formulations mentioned in this review, including Xianling Gubao Capsule and Jiangu Granule. The clinical evidence for these formulations remains limited to traditional bone-related endpoints without integrated GM analysis, highlighting a critical translational gap. Therefore, the clinical claims regarding CBD efficacy in OP management remain provisional, and the lack of human data severely limits the direct applicability of current findings; ③ research is limited to single signaling pathways (e.g., SCFA-Wnt/β-catenin, TLR4/MyD88/NF-κB), lacking systematic analysis of the multi-level, multi-pathway synergistic regulation of the gut-bone axis by CBDs and the key crosstalk nodes between microbial metabolites, endocrine factors and immune cells in the bone microenvironment; ④ GM-mediated CBD biotransformation research is in its infancy, with uncharacterized specific intestinal bacteria, metabolite structures and their subsequent bone metabolic effects; ⑤ long-term safety profiles of CBDs, including potential hepatotoxicity, herb-drug interactions, and risks of GM dysbiosis with prolonged use, remain poorly characterized (Table 3), and non-standardized GM detection methods lead to poor reproducibility across studies.

**TABLE 4:** Potential risks, herb-drug interactions, and gut dysbiosis of individual CBDs.

CBDs	Potential risks	Herb-drug interactions	Long-term use risk of gut dysbiosis
Berberine	GI disturbance, diarrhea, abdominal pain; mild hypotension; potential cardiac effects at high doses	Anticoagulants (↑bleeding); antidiabetics (↑hypoglycemia); CYP450 inhibitors	Reduces gut microbial diversity; inhibits commensal bacteria; depletes SCFA-producing bacteria; disrupts F/B ratio
Puerarin	Dizziness, nausea, mild hypotension; skin rash; weak estrogen-like activity	Antihypertensives (excessive BP drop); anticoagulants/antiplatelets (↑bleeding)	Alters F/B ratio; suppresses beneficial genera; reduces GM stability; impairs SCFA production
Lignans	Mild nausea, gastrointestinal discomfort; rare hypersensitivity	Anti-inflammatory drugs; anticoagulants	Disrupts tryptophan-metabolizing flora; reduces microbial richness and diversity
Loganin	Mild GI upset; rare allergic reactions	No obvious severe interactions; may enhance analgesic/anti-inflammatory effects	Mildly decreases microbial richness; may reduce Bifidobacterium and *Lactobacillus*
Oleanolic Acid	Mild hepatotoxicity at high doses; GI discomfort; dizziness	Hepatotoxic drugs (↑liver risk); anticoagulants; hypoglycemic agents	Inhibits commensal flora; increases pro-inflammatory taxa; impairs gut barrier-related microbiota
Total flavonoids *Eucommia ulmoides Oliv. leaves*	Mild drowsiness, GI upset, hypotension	Antihypertensives; sedatives; anticoagulants	Disturbs F/B ratio; reduces SCFA-producing bacteria; weakens intestinal colonization resistance
Astragalus polysaccharides (APS)	Bloating, diarrhea, flatulence; may over-activate immune response	Immunosuppressants (antagonism); antidiabetics; antihypertensives	Induces abnormal flora overgrowth; reduces GM diversity; impairs metabolic function of microbiota
Water extract of *Epimedium brevicornu Maxim*	Dizziness, palpitations, dry mouth; estrogen-like effects; uterine stimulation	Hormone therapies; antihypertensives; anticoagulants	Inhibits commensal flora; disrupts Th17/Treg-related microbiota; reduces GM resilience
Water extract of *Ligustrum lucidum* *Ait*	Bloating, diarrhea; skin rash; mild liver enzyme elevation at high dose	Hepatotoxic drugs; hypoglycemics; diuretics	Inhibits anaerobic commensals; reduces Bifidobacterium; impairs intestinal barrier function
Morinda officinalis How polysaccharides	Dryness, thirst, mild constipation; warm-natured may cause internal heat	Anticoagulants; antihypertensives; sedatives/hypnotics	Alters GM structure; reduces *Lactobacillus*/Bifidobacterium; impairs SCFA generation
Gegen Qinlian Decoction (GGQLD)	Dry mouth, diarrhea, abdominal pain; bitter-cold property may damage spleen	Antibiotics; anticoagulants; hypoglycemics; CYP450 substrates	Strong antibacterial effects; kills commensals; induces long-term dysbiosis; reduces diversity
Tenghuang Jiangu pill (THJGP)	Mild GI discomfort; dry mouth; dizziness	Anticoagulants; antihypertensives; anti-inflammatory drugs	Disturbs F/B ratio; reduces probiotic abundance; impairs gut immune-related microbiota
Xianling Gubao capsule (XLGBC)	Estrogen-like effects; GI upset; dizziness; risk of drug-induced liver injury	Anticoagulants; antihypertensives; hormone therapies; SERMs	Multi-component disturbance; reduces GM diversity; depletes SCFA-producing bacteria
Yishen Zhuanggu decoction (YSZGD)	Bloating, nausea; warm-tonifying may cause excessive internal heat	Anticoagulants; immunosuppressants; hormone drugs	Alters GM composition; increases conditional pathogens; reduces colonization resistance
Jiangu granule (JGG)	GI discomfort; mild rash; abdominal bloating	Antidiabetics; antihypertensives; anticoagulants	Imbalances F/B ratio; disrupts SCFA–Treg/Th17 axis flora; reduces GM stability
Zhuanggu Zhitong capsule (ZGZTC)	Dizziness, GI upset; estrogen-like effects	Anticoagulants; antihypertensives; hormone preparations	Reduces microbial diversity; increases pro-inflammatory taxa; impairs gut barrier
Shengu granule (SGG)	Bloating, diarrhea; mild allergic reactions	Antidiabetics; immunosuppressants; anticoagulants	Disturbs commensal balance; depletes SCFA producers; may induce secondary dysbiosis
Bushen Jianpi formula (BSJPF)	Bloating, stomach distension; rare skin rash	Immunosuppressants; hormones; diuretics	Promotes conditional pathogen overgrowth; reduces GM diversity and resilience
Bushen Huoxue decoction (BSHXD)	Mild GI upset; dry mouth; dizziness	Anticoagulants; antiplatelets; antihypertensives	Disrupts Firmicutes/Proteobacteria ratio; impairs mucosal flora; reduces diversity

To address these limitations and advance CBD-based OP therapy via the gut-bone axis, future research should focus on the following key directions, combining TCM theory with modern molecular biology, microbiology and clinical research: ① Systems/network pharmacology for multi-component CBD synergism: Utilize multi-omics (metagenomics, metabolomics, transcriptomics) and bioinformatics to construct the “CBD active metabolites-GM-bone metabolism” regulatory network, identifying core active ingredients and their holistic regulatory mechanisms on the gut-bone axis; ② Humanized and diverse OP model development: Establish complex models mimicking human OP pathologies (e.g., aged humanized GM mice, OP models with comorbidities like diabetes) and integrate *in vitro* models (intestinal organoids, bone cell co-cultures) to provide a more reliable experimental basis for clinical translation; ③ Multi-omics integration to dissect gut-bone axis crosstalk: Combine metagenomics, single-cell RNA sequencing and spatial transcriptomics to analyze GM, microbial metabolite and gene/protein expression changes in intestinal and bone microenvironments after CBD intervention, clarifying key regulatory factors and signaling pathways; ④ in-depth exploration of CBD-GM bidirectional interaction: Characterize intestinal bacteria/enzymes involved in CBD biotransformation and the activity of generated metabolites, while exploring GM’s effects on CBD absorption and metabolism; develop probiotic/prebiotic-modified CBD formulations to enhance GM-targeting effects and bioavailability; ⑤ Large-scale clinical research and biomarker validation: Conduct well-designed randomized controlled trials to evaluate CBD efficacy/safety in OP treatment, with particular emphasis on including GM-related endpoints (e.g., metagenomic analysis, fecal SCFA quantification, intestinal barrier function assessment) alongside conventional bone outcome measures (BMD, serum CTX-1, P1NP, OCN), and establish human OP cohorts to validate GM biomarkers for OP diagnosis, prognosis and personalized therapy, providing high-quality clinical evidence; ⑥ Novel GM-targeted anti-OP therapy development: Develop CBD-probiotic/prebiotic composite preparations and combine CBDs with conventional anti-OP drugs to achieve synergistic effects and reduce adverse reactions; ⑦ optimize CBD dosage forms to enhance intestinal targeting and patient compliance.

## 6 Conclusion

In conclusion, the gut-bone axis acts as a pivotal regulatory network underlying the pathogenesis of osteoporosis. CBDs exert prominent anti-osteoporotic effects primarily by reshaping GM composition, repairing intestinal barrier integrity, and rebalancing metabolic, endocrine, and immune signaling within the gut-bone axis. This review confirms the causal relationship between GM dysbiosis and bone loss, illustrates the bidirectional interaction between CBDs and GM, and identifies valuable microbiota-related biomarkers for bone health. Collectively, these insights establish a theoretical foundation for developing novel GM-targeted therapeutic strategies and accelerating the clinical translation of CBDs in OP management.
