# Supplementary material for: Gut-bone axis: mechanisms and intervention effects of Chinese botanical drugs in osteoporosis management
Source: Front Pharmacol. 2026 Jun 17;17:1842067. doi: 10.3389/fphar.2026.1842067 (PMC13318907; doi:10.3389/fphar.2026.1842067)
Supplement: Supplementary file 1 [file Supplementaryfile1.docx]

**Literature search strategy**

The research questions are as follows: (1) How does gut microbiota dysbiosis manifest in osteoporosis and correlate with bone metabolic abnormalities? (2) What are the key mechanistic pathways (metabolite, endocrine, and immune) through which gut microbiota dysbiosis drives osteoporosis development? (3) Which Chinese botanical drugs and their active metabolites have demonstrated anti-osteoporotic effects specifically by targeting the gut-bone axis, and what are their underlying molecular and microbial mechanisms? The prospective protocol for this review was pre-established, with search terms and inclusion/exclusion criteria carefully selected to ensure comprehensive coverage of all relevant literature. A systematic literature search was performed on January 5, 2026, across the following electronic databases: Web of Science (n =86), PubMed (n =23), Embase (n=13), and China National Knowledge Infrastructure (CNKI) (n =140).

The search strategy employed free-text terms using the following keywords and Boolean operators:

(“Gut microbiota” OR “Gut microflora” OR “Gastrointestinal microbiome” OR “Microbiome, Gastrointestinal” OR “Enteric microbiota” OR “Intestinal flora”) AND (“Osteoporosis” OR “Osteoporosis, Age-Related” OR “Age-Related Osteoporosis” OR “Bone Loss, Age-Related” OR “Bone Losses, Age-Related” OR “Osteoporosis, Senile” “Osteoporoses, Senile” OR “Senile Osteoporoses” OR “Senile Osteoporosis”) AND (“Chinese botanical drug” OR “Chinese traditional medicine” OR “medicine, Chinese traditional” OR “Chinese herbal medicine” OR “Chinese traditional medicine” OR “medicine, Chinese traditional” OR “Chinese Drugs, Plant” OR “Chinese Herbal Drugs” OR “Extracts, Chinese Plant”). External limitations were applied to each database, though specific constraints varied due to differences in search options.

No language restrictions were applied, but the search was primarily limited to studies published within the last 10 years to ensure currency of the findings. A limited number of older references of significant relevance were also included. The search strategies were adapted as necessary to accommodate the specific search interfaces and syntax requirements of each database.

**Inclusion criteria** were: (1) original research articles (in vitro, in vivo, or clinical studies) or peer-reviewed articles; (2) studies investigating the regulatory role of the gut microbiota or its metabolites on bone metabolism; (3) studies on the anti-osteoporotic effects of Chinese botanical drugs or their active metabolites involving gut microbiota modulation.

**Exclusion criteria** were: (1) studies not related to the gut-bone axis, gut microbiota, osteoporosis or Chinese botanical drugs (n =11); (2) Reviews, meta-analyses, conference abstracts, letters, editorials, and expert opinions (n=45); (3) Studies with unclear experimental design, incomplete data, or inconsistent outcome indicators (n=3). Ultimately, 57 studies were included in this review due to their direct relevance to the gut-bone axis and Chinese botanical drug interventions.

**Literature screening and data extraction**

All retrieved records were imported into reference management software for deduplication. HL and SL independently screened the titles and abstracts of the remaining records against the eligibility criteria. Full texts of potentially relevant studies were then retrieved and assessed independently by the same two reviewers. Disagreements were resolved through discussion or consultation with a third reviewer.

Data were extracted from the included studies, focusing on the regulatory mechanisms of the gut microbiota and its metabolites in osteoporosis, as well as the therapeutic effects and targets of Chinese botanical drugs. A detailed screening flowchart is presented in **Figure 1**.

**Identification of studies via database**

Records identified from Databases (n = 262)

**Identification**

Records after duplicates removed (n =221)

Records excluded (n = 111)

Records screened (n = 110)

**Screening**

Full-text articles excluded, with reasons:

(1) studies not related to the gut-bone axis, gut microbiota, osteoporosis or Chinese botanical drugs (n =11);

(2) Reviews, meta-analyses, conference abstracts, letters, editorials, and expert opinions (n=45);

(3) Studies with unclear experimental design, incomplete data, or inconsistent outcome indicators (n=3).

Full-text articles assessed for eligibility (n = 57)

Articles included in review (n = 57)

**Included**

**Figure 1 Flowchart of the article selection process**

A total of 262 articles were initially identified from PubMed (n=23), Web of Science (n=86), Embase (n=13), and China National Knowledge Infrastructure (CNKI) (n=140). After removing duplicates, 221 records were screened. Following title and abstract screening, 170 full-text articles were assessed for eligibility. Exclusion criteria applied at the full-text stage included: (1) studies not related to the gut-bone axis, gut microbiota, osteoporosis or Chinese botanical drugs (n =11); (2) Reviews, meta-analyses, conference abstracts, letters, editorials, and expert opinions (n=45); (3) Studies with unclear experimental design, incomplete data, or inconsistent outcome indicators (n=3). Ultimately, 57 studies met the inclusion criteria and were included in the review.
